# Checkpoint inhibition in hematologic malignancies

**DOI:** 10.3389/fonc.2023.1288172

**Published:** 2023-10-17

**Authors:** Aaron Tsumura, Daniel Levis, Joseph M. Tuscano

**Affiliations:** ^1^ Division of Malignant Hematology/Cellular Therapy and Transplantation, University of California Davis, Sacramento, CA, United States; ^2^ School of Medicine, University of California Davis, Sacramento, CA, United States

**Keywords:** hematologic malignancies, leukemia, lymphoma, checkpoint inhibitors, immunotherapy, cd47, LAG-3, TIGIT

## Abstract

Checkpoint inhibitor therapy has emerged as an effective therapeutic strategy for many types of malignancies, especially in solid tumors. Within the last two decades, numerous monoclonal antibody drugs targeting the CTLA-4 and PD-1/PD-L1 checkpoint pathways have seen FDA approval. Within hematologic malignancies, Hodgkin Lymphoma has seen the greatest clinical benefits thus far with more recent data showing efficacy in the front-line setting. As our understanding of checkpoint inhibition expands, using these pathways as a therapeutic target has shown some utility in the treatment of other hematologic malignancies as well, primarily in the relapsed/refractory settings. Checkpoint inhibition also appears to have a role as a synergistic agent to augment clinical responses to other forms of therapy such as hematopoietic stem cell transplant. Moreover, alternative checkpoint molecules that bypass the well-studied CTLA-4 and PD-1/PD-L1 pathways have emerged as exciting new therapeutic targets. Most excitingly is the use of anti-CD47 blockade in the treatment of high risk MDS and TP-53 mutated AML. Overall, there has been tremendous progress in understanding the benefits of checkpoint inhibition in hematologic malignancies, but further studies are needed in all areas to best utilize these agents. This is a review of the most recent developments and progress in Immune Checkpoint Inhibition in Hematologic Malignancies in the last decade.

## Introduction

One of the primary hallmarks of malignancy is a tumor cell’s ability to resist cell death (1). The inhibitory molecules cytotoxic T lymphocyte-associated protein (CTLA-4) and programmed cell death protein 1 and its ligand (PD-1 and PD-L1, respectively) are well-studied signaling molecules that serve in controlling T-cell activation, maturation, and viability. CTLA-4 on the naïve T-cell membrane competes with CD28 to bind CD80 on the antigen presenting cell (APC) and is thought to regulate T-cell function early in the immune response (2). PD-1 on the activated T-cell binds its ligand PD-L1 on the APC and represses stimulatory signaling, leading to anergy, apoptosis, and differentiation to Regulatory T-cells (Tregs). This interaction occurs later in the immune response and within peripheral tissues (2, 3). By targeting these pathways and inhibiting suppressive immune checkpoint effects, Immune Checkpoint Inhibitors (ICI) has proven to be effective for many hematologic malignancies both in the front-line and relapse/refractory setting. ICI therapy has also shown benefit as adjunctive therapy with other treatment modalities such as Hematopoietic Stem Cell Transplant, Chimeric Antigen Receptor T-Cell Therapy, and Radiation Therapy. More recently, newer immune checkpoint molecules have been discovered and serve as promising alternative targets for ICI in Hematologic Malignancies as well. This is a comprehensive review of the current state of ICI in Hematologic Malignancies.

## Hodgkin lymphoma

Of all hematologic malignancies, Hodgkin Lymphoma (HL) has seen the largest benefit in ICI-based therapy thus far, especially in the relapsed/refractory (r/r) setting. The major immunosuppressive effector cells present in the Tumor Microenvironment (TME) of HL appear to be exhausted PD-1^+^ Th1 effector cells and active PD-1^-^ Th1 Tregs (4). The primary malignant cell, the Reed-Sternberg cell, also has higher PD-L1 expression and lower Major Histocompatibility Complex-1 (MHC1) expression, while also having a higher concentration of surrounding CTLA-4^+^ CD4 T-cells (4, 5). This is consistent with existing data supporting the benefit of ICI therapy for HL as highlighted in **Table 1**. Both the KEYNOTE-013 and KEYNOTE-087 trials have led to the approval of Pembrolizumab while the CheckMate 205 trial led to the approval of Nivolumab for the treatment of r/r HL after multiple lines of therapy, including after autologous hematopoietic stem cell transplant (autoHCT). Though overall response rates have been reported between 60-70%, most patients still relapse (6–8). Further systematic reviews assessing PD-1/PD-L1 inhibitors in r/r HL have measured a composite Objective Response Rate (ORR) of 79%, Complete Response (CR) of 44%, and Partial Response (PR) of 34% (9). The KEYNOTE-204 trial showed a median Progression Free Survival (PFS) of 13.2 months with Pembrolizumab salvage therapy for r/r HL after autoHCT (10). Additionally, patients with r/r HL who were treated with Nivolumab saw an ORR of 70% with a CR rate of 43.3% and median PFS of 18.4 months (11). Thus, ICI has shown remarkable efficacy in the treatment in r/r HL with reported ORR ranging between 60-70%, CR ranging between 40-45%, and PFS ranging between 13-18 months, even after autoHCT.

**Table 1:** Recent published trials evaluating CPI in HL.

Name/Code	Year	Phase	Disease/ Status	Sample Size	Intervention	Response	Duration	Ref #
KEYNOTE-013	2016	IB	R/R cHL	31	Pembro	ORR: 65% CR: 16% PR: 48%	24-wk PFS: 69% 52-wk PFS: 46%	(6)
KEYNOTE-087	2017	II	R/R HL	210	Pembro	ORR: 69.0% CR: 22.4%	DOR ≥ 6 mo: 31%	(7)
CheckMate 205	2018	II	R/R cHL	243	Nivo	ORR: 69%	mDOR: 16.6 mo mPFS: 14.7 mo	(8)
Meta-analysis	2023	n/a	R/R HL	1440	PD-1/PD-L1 blockade	pooled ORR: 79% pooled CR: 44% pooled PR: 34% Cumulative: mOS: not reached 2-yr OS: 89.8%	Nivo monotherapy mPFS: 13.7 mo mDOR: 17.3 mo 2-yr PFS: 33.5% 2-yr DOR: 26.6% Pembro monotherapy mPFS: 17.2 mo mDOR: 18.4 mo 2-yr PFS: 38.4% 2-yr DOR: 43.2%	(9)
KEYNOTE-204	2021	III	R/R cHL	304	Pembro vs BV	Not reported	Pembro vs BV mPFS: 13.2 mo vs 8.3 mo	(10)
NCT03343665	2020	II	R/R cHL	30	Nivo	ORR: 70% CR: 43.3% mOS: not reached	mPFS: 18.4 mo 18-mo PFS: 53.6%	(11)
PMID: 32601377	2020	IB	R/R cHL R/R NHL R/R MM	137	Nivo/Ipi vs Nivo/Liri	(cHL) Nivo/Ipi vs Nivo/Liri ORR: 74% vs 76% CR: 23% vs 24%	mPFS: not reached	(12)
NCT02572167	2021	I/II	R/R cHL	93	Nivo + BV as first salvage	ORR: 85% CR: 67% 3-yr OS: 93%	3-yr PFS no HCT: 77% 3-yr PFS with HCT: 91%	(13)
NCT02665650	2020	IB	R/R HL	30	Pembro + AFM13	All doses ORR: 83% CR: 37% PR: 47% Highest dose ORR: 88% CR: 42% PR: 46%	All doses: mDOR (all): 9.9 mo mDOR (CR): 10.4 mo mDOR (PR): 9.0 mo Highest dose: mDOR (all): 9.0 mo mDOR (CR): 10.4 mo mDOR(PR): 8.7 mo	(14)
ACCRU	2020	II	Elderly or unfit HL	46	Nivo + BV as first-line	ORR: 61% CR: 48% PR: 13% mOS: not reached	mPFS: 18.3 mo mPFS (CR): not reached	(15)
NIVAHL	2020 2023	II	ES-cHL	109	Nivo + AVD	Concomitant vs Sequential ORR: 100% vs 96% CR: 90% vs 94% 41-mo OS: 100% vs 100%	Concomitant vs Sequential 12-mo PFS: 100% vs 98% 41-mo PFS: 100% vs 98%	(16, 17)
SWOG S1826	2023	III	AS-cHL	994	Nivo+AVD vs BV+AVD	Response rates pending	Nivo+AVD vs BV+AVD 1-yr PFS: 94% vs 86%	(18)
PMID: 36497328	2022	n/a	R/R HL	26	Anti-PD1 consolidation following autoHCT	2-yr OS: 87%	mPFS: 42.6 mo 2-yr PFS: 79%	(19)
NCT03057795	2023	II	R/R cHL	59	Nivo + BV after autoHCT	24-mo OS: 98%	18-mo PFS: 94% 24-mo PFS: 92%	(20)

R/R Relapsed/Refractory, cHL Classical Hodgkin Lymphoma, Pembro Pembrolizumab, Nivo Nivolumab, ORR Objective/Overall Response Rate, CR Complete Response, PR Partial Response, PFS Progression Free Survival, DOR Duration of Response, wk Week, mo Month, yr Year, BV Brentuximab vedotin, Ipi Ipilimumab, Liri Lirilumab, AVD Doxorubicin + Vinblastine + Dacarbazine, autoHCT Autologous Hematopoietic Stem Cell Transplant.

AFM13 (bispecific anti-CD30/CD16A antibody).

While ICI monotherapy proves effective in the r/r setting, two critical knowledge gaps remain: whether ICI when used in combination therapies add additional outcome improvements and the optimal timing of ICI in relation to autoHCT. Armand et al. concluded that the addition of anti-CLTA-4 or anti-KIR to Nivolumab in treating r/r Classical HL (cHL) after autoHCT did not add benefit, but rather had higher rates of Treatment-Related Adverse Events (TRAEs). They report that though ORRs of the combinations Nivolumab/Ipilimumab (Nivo/Ipi) and Nivolumab/Lirilumab (Nivo/Liri) were 74% and 76%, respectively, rates of TRAE were 29% and 15%; higher than reported for Nivolumab alone (12). This data suggests no additional benefit to ICI use in combination therapies after autoHCT. Alternatively, there appears to be improved PFS rates when ICI is used as consolidative therapy with autoHCT, both as monotherapy and in combination. Filippi et al. report a median PFS of 42.6 months, 2-year PFS of 79%, and 2-year OS of 87% with consolidative Nivolumab or Pembrolizumab in high-risk HL with salvage autoHCT (19). Additionally, Herrera et al. report an 18-month PFS of 94% with consolidative combination Nivolumab with Brentuximab Vedotin after autoHCT (20). Thus this data would suggest a PFS benefit in using ICI as consolidative therapy following autoHCT. Furthermore, positive data also suggests improved outcomes with ICI-combination as first salvage therapy before autoHCT. Advani et al. report an 85% ORR, 67% CR, and 3-year PFS of 77% with combination Nivolumab with Brentuximab Vedotin as first salvage therapy for r/r cHL. They further report that patients who proceeded to autoHCT had an improved 3-year PFS to 91% and 3-year OS of 93% (13). Bartlett et al. reported a secondary endpoint of an 88% ORR in a heavily pretreated patient population treated with a combination of Pembrolizumab with a bispecific anti-CD30/CD16A antibody (AFM13) (14). Thus, current available data would suggest that ICI-combination therapy has greater outcome benefits when used either before autoHCT or as a consolidative regimen with autoHCT, but provides no further benefit as compared to ICI monotherapy when used as salvage therapy after autoHCT.

ICI-combination therapies are also being evaluated as first-line therapy and have shown very promising improvement in outcomes. The ACCRU trial evaluated Nivolumab plus Brentuximab Vedotin as first-line therapy for older and chemotherapy-ineligible HL patients. Although the trial was closed due to failure to meet predefined criteria, analysis of the 46 enrolled patients showed a CR rate of 48%, and PR of 13%, giving an ORR of 61%. This suggests that although combination immunotherapy may be active in older patients and patients with comorbidities, further dosing and scheduling optimization is needed (15). More significantly, the NIVAHL trial evaluated Nivolumab in combination with Doxorubicin, Vinblastine, and Dacrabazine (N-AVD) as first-line therapy for early-stage unfavorable cHL. Patients with newly diagnosed cHL were randomized to 4 cycles of concomitant N-AVD or sequentially administered 4 doses of Nivolumab followed by 2 cycles of N-AVD followed by 2 cycles of AVD alone. Each group then received 30-Gy radiation to affected sites. Initial analysis published in 2020 reported CR rates of 90% for the concomitant group and 94% for the sequential group. 12-month PFS was 100% for the concomitant group and 98% for the sequential group (16). The investigators reported their final analysis in early 2023 showing that at a median follow-up of 41 months, OS was 100% in both groups. PFS rate at last follow-up remained 100% and 98% for the concomitant and sequential groups, respectively (17). A separate analysis of the study population showed a sustained decrease in exhausted T-cell phenotype in patients receiving anti-PD1 therapy (21). Furthermore, the SWOG S1826 trial compared N-AVD to Brentuximab vedotin-AVD (BV-AVD) as first-line in advanced stage cHL. Patients with stage 3-4 HL were randomized 1:1 to either 6 cycles of N-AVD or BV-AVD. 12-month PFS was significantly superior in the N-AVD arm at 94% vs 86% for the BV-AVD arm. 11 deaths were observed in the BV-AVD arm compared to 4 in the N-AVD arm (18). Longer follow-up is needed to assess OS, but with preliminary data showing response rates and OS rates ranging from 90-100%, this data is anticipated to be practice-changing.

Data thus far has shown that ICI therapy is remarkably effective in nearly all treatment settings of HL. A list of active studies is shown in **Tables 2A**, **B** evaluating ICI activity in both the front-line and relapsed/refractory settings, respectively. There have been an abundance of data supporting the efficacy and safety of ICI therapy in the treatment of r/r HL, especially after autoHCT. More recent trials have shown significant benefits when utilizing ICI therapy prior to autoHCT and as consolidative therapy with autoHCT. Most exciting, is data supporting ICI-combination as front-line therapy as shown with the addition of Nivolumab to upfront AVD for both early-stage and advanced-stage cHL, which could potentially change our front-line treatment paradigm.

**Table 2A:** Current clinical trials evaluating CPI in first-line treatment of HL.

NCT Number	Phase	Disease Focus	Intervention
NCT03033914	I/II	HL	Nivo + ABVD
NCT03598608	I/II	cHL, NHL	Pembro + Favezelimab
NCT03407144 (KEYNOTE 667)	II	AYA cHL	Pembro + first-line SOC Chemotherapy
NCT03712202	II	Early-stage cHL	Nivo + BV
NCT03233347	II	Early-stage HL	Nivo + BV + AVD
NCT05900765	II	Early-stage HL	Zimberelimab + AVD
NCT03331731 (PLIMATH)	II	HL ineligible for chemo	Pembro monotherapy
NCT05008224 (KEYNOTE-C11)	II	cHL	Pembro followed by first-line SOC Chemotherapy
NCT03226249	II	cHL	Pembro + AVD
NCT03331341	II	cHL	Pembro + AVD
NCT05772624	II	cHL	Nivo + AVD
NCT03617666	II	Advanced Stage HL	Avelumab monotherapy
NCT03580408	II	Elderly cHL	Nivo +/- Vinblastine
NCT05404945	II	Elderly cHL	Pembro + BV
NCT02758717	II	Elderly HL	Nivo + BV

**Table 2B:** Current clinical trials evaluating CPI in r/r HL.

NCT Number	Phase	Disease Focus	Intervention
NCT03681561	I	R/R cHL	Nivo + Ruxolitinib
NCT02408861	I	R/R HIV-cHL	Nivo + Ipi
NCT05162976	I	R/R HL after prior CPI	Nivo + AZA
NCT05352828	I	R/R cHL	Nivo with CAR-T
NCT04134325 (LCCC1852-ATL)	I	R/R cHL	Nivo or Pembro after CAR-T
NCT05255601 (RELATIVITY-069)	I/II	AYA R/R cHL	Nivo + Relatlimab
NCT03739619	I/II	R/R cHL	Nivo + Gemcitabine + Bendamustine
NCT04981899	I/II	R/R HL	Nivo + ICE
NCT03343652	I/II	R/R HL	Nivo + Bendamustine
NCT03436862	II	HR-HL	Nivo maintenance after autoHCT
NCT03057795	II	HR-cHL	Nivo + BV consolidation after autoHCT
NCT03016871	II	R/R HL	Nivo + ICE as second line
NCT04091490 (Nivo-DHAP-cHL)	II	R/R HL	Nivo + DHAP
NCT05660993	II	R/R HL	Nivo + BeGEV
NCT03337919 (ANIMATE)	II	R/R HL	Nivo monotherapy as bridge to HCT
NCT03618550	II	R/R HL	Pembro + GVD as second line
NCT03179917	II	R/R HL	Pembro + Radiation Therapy
NCT04510636	II	R/R HL	Pembro + Bendamustine
NCT03077828	II	R/R HL	Pembro + ICE
NCT05355051	II	R/R HL	Pembro + AZA
NCT05039073	II	R/R HL after prior CPI	Nivo + BV
NCT03480334	II	R/R HL after prior CPI	Nivo + Radiation Therapy
NCT01703949	II	R/R HL, R/R NHL	BV +/- Nivo
NCT02927769	II	AYA R/R cHL	Nivo + BV
NCT04938232	II	R/R cHL	Ipi +/- Nivo
NCT02940301	II	R/R cHL	Nivo + Ibrutinib
NCT04561206	II	R/R cHL	Nivo + BV
NCT05723055	II	R/R cHL	Nivo + Axatilimab
NCT02453594 (KEYNOTE-087)	II	R/R cHL	Pembro monotherapy
NCT04788043	II	R/R cHL	Pembro + Magrolimab
NCT05180097	II	R/R cHL	Pembro + BV vs GDP
NCT04875195	II	R/R cHL, R/R PMBCL	Pembro monotherapy
NCT05595447	II/III	R/R HL	BV + anti-PD-1 before and after AutoHCT
NCT03907488 (SWOG S1826)	III	Advance Stage cHL	Nivo + AVD vs BV + AVD
NCT05675410	III	Early-stage cHL	Nivo + BV + first-line SOC vs SOC
NCT02684292	III	R/R cHL	Pembro vs BV
NCT05508867 (KEYFORM-008)	III	R/R cHL	Pembro/Favezelimab vs physician’s choice

cHL Classical Hodgkin Lymphoma, Nivo Nivolumab, ABVD Doxorubicin + Bleomycin + Vinblastine + Dacarbazine, NHL Non-Hodgkin Lymphoma, Pembro Pembrolizumab, AYA Adolescent and Young Adult, SOC Standard of Care, BV Brentuximab vedotin, R/R Relapsed/Refractory, HIV-cHL HIV-associated cHL, Ipi Ipilimumab, AZA Azacitidine, CPI Checkpoint Inhibitor, CAR-T Chimeric Antigen Receptor T-cell, ICE Ifosfamide + Carboplatin + Etoposide, HR-HL High-Risk HL, autoHCT Autologous Hematopoietic Stem Cell Transplant, DHAP Dexamethasone + Cytarabine + Cisplatin, BeGEV Bendamustine + Gemcitabine + Vinorelbine, GVD Gemcitabine + Vinorelbine + Doxorubicin, GDP Gemcitabine + Dexamethasone + Cisplatin.

## Non-Hodgkin lymphoma

Non-Hodgkin Lymphomas (NHL) have seen some benefit to ICI therapy, though not as robust as in HL, which is thought, in part, to be due to a differing milieu of tumor infiltrating immune cells. Diffuse Large B-Cell Lymphoma (DLBCL) is known to have lower expression of CD3^+^ and CD4^+^ tumor infiltrating lymphocytes (TILs) as well as lower PD-L1 expression as compared to HL (22). Studies have also shown that a higher ratio of CD4^+^/CD8^+^ T-cells to PD-L1 tumor expression is an independent predictor of better 4-year OS of DLBCL patients treated with Standard of Care (SOC) chemotherapy of Rituximab, Cyclophosphamide, Doxorubicin, Vincristine, Prednisone (R-CHOP) (23, 24). This supports the idea that PD-L1 expression serves an important role in immune evasion and tumor proliferation while being an important predictive measure in responsiveness to ICI-based therapies. Reported ORRs of r/r DLBCL to ICI either as monotherapy or in combination therapy range from 9-20%. Alternatively, ORRs for r/r Follicular Lymphoma (FL) have been more variable and range from 10-60% while KEYNOTE-013 reported an ORR of 48% for r/r Primary Mediastinal B-cell Lymphoma (PMBL) (12, 25–27). Thus, the variable response rates in NHLs serve as basis for further optimization of ICI use in the treatment of these malignancies.

Newer data suggests a potential role in treating Richter Transformation-DLBCL (RT-DLBCL). Studies have shown an upwards of 40% of RT-DLBCL tumors have >50% PD-1/PD-L1 expression, and >60% of tumors have >20% PD-1^+^ TILs. These patients also tended to have significantly better OS compared to those who had lower PD-1+ TILs (28). Jain et al. saw an ORR of 42% with a median Duration of Response (DOR) of 15 months in RT-DLBCL patients treated with Nivolumab and Ibrutinib. Median OS was 13 months (29). More notably, Herrera et al. evaluated the addition of Atezolizumab to immunogenic chemoimmunotherapy, Rituximab with Gemcitabine and Oxaliplatin (R-GEMOX) in treating RT-DLBCL from either B-NHL or Chronic Lymphocytic Leukemia (CLL). Patients who achieved CR after 6 doses of R-GEMOX with Atezolizumab went on to Rituximab with Atezolizumab maintenance for up to 2 years. The ORR was 50% with a CR rate of 29%. Of the patients who achieved CR, 2 went on to HCT while the other 4 had ongoing CR without additional therapy lasting between 5-30 months. The investigators also noted better responses to R-GEMOX with Atezolizumab in RT-DLBCL from B-NHL as compared to traditional Richter Transformation from CLL (30).

Summarized in **Table 3**, the benefits of the addition of ICI agents Nivolumab, Pembrolizumab, and Durvalumab for B-cell NHLs have been variable, possibly related to the variability in CD3^+^/CD4^+^ TILs and tumor PD-L1 expression. Interestingly, Atezolizumab appears to provide significant benefit when combined with R-GEMOX in the treatment of RT-DLBCL. Thus, further studies are required to optimize ICI-based therapies in B-cell NHLs.

**Table 3:** Recent published trials evaluating CPI in NHL.

Name/Code	Year	Phase	Disease/Status	Sample Size	Intervention	Response	Duration	Ref #
PMID: 32601377	2020	IB	R/R cHL R/R NHL R/R MM	137	Nivo/Ipi vs Nivo/Liri	NHL Nivo/Ipi or Nivo/Liri ORR: 9-22% CR: 0-6%	mPFS: not reached	(12)
KEYNOTE-155	2022	IB	R/R DLBCL	38	Pembro + Dinaciclib	ORR: 21.1%		(25)
KEYNOTE-013	2023	IB	R/R NHL	89	Pembro	ORR (overall): 22% ORR (PMBCL): 48% ORR (FL): 10% ORR (DLBCL): 15%		(26)
FUSION NHL 001	2023	I/II	R/R NHL	106	Durvalumab monotherapy or in combination	ORR (FL): 59% ORR (DLBCL): 18%		(27)
NCT02420912	2023	II	RT-DLBCL	24	Nivo + Ibrutinib	ORR: 42%	mDOR: 15 mo mOS: 13 mo	(29)
R-GemOx+Atezo	2021	I	R/R RT-DLBCL	23	R-GemOx + Atezolizumab	ORR: 50% CR: 29%	mDOR: not reached in responders	(30)

R/R Relapsed or Refractory, cHL Classical Hodgkin Lymphoma, NHL Non-Hodgkin Lymphoma, MM Multiple Myeloma, Nivo Nivolumab, Ipi Ipilimumab, Liri Lirilumab, ORR Objective Response Rate, CR Complete Response, mPFS Median Progression Free Survival, DLBCL Diffuse Large B-Cell Lymphoma, Pembro Pembrolizumab, FL Follicular Lymphoma, RT-DLBCL Richter Transformation-DLBCL, mDOR Median Duration of Response, mOS Median Overall Survival, R-GemOx Rituximab + Gemcitabine + Oxaliplatin.

Currently active studies are evaluating Ipilimumab as potential ICI therapy in r/r B-cell NHLs (NCT00089076). There is also particular interest in taking advantage of the immunomodulatory effects of ICI to sensitize tumors to other therapies. NCT03533283 is an open-label Phase IB/II study evaluating the safety and efficacy of the CD20/CD3 bispecific antibody, Glofitamab, in combination with Atezolizumab or Polatuzumab Vedotin for adults with r/r B-NHLs. This combination is intriguing whereby blocking inhibitory Tregs may allow for the activation of tumor-specific effector T-cells via anti-PD-1 blockade. Atezolizumab would thus augment the effects of Glofitamab. Another intriguing therapeutic approach is intratumoral injection of anti-CTLA-4 ICI. NCT01769222 sets to evaluate the potential sensitizing effects of intratumoral Ipilimumab to local radiation therapy in a number of solid tumors, including NHLs that would increase neoantigen expression and expand the immune response (aka abscopal effect). In summary, ICI-based therapy has provided variable benefits on B-cell NHLs in which further investigation is needed to fully maximize its therapeutic effectiveness, which may include using ICI-based therapy as a synergistic augmentation to other effective therapies for NHLs.

## Primary CNS lymphoma

Primary Central Nervous System Lymphoma (PCNSL) is a particular form of extra-nodal NHL that originates from tissue of the central nervous system (i.e. brain, spinal cord, eye, and meninges) and has been associated with Epstein-Barr Virus reactivation (EBV) as well as underlying immune deficiency (31, 32). First-line therapy is usually high-dose methotrexate-based regimens and though most will respond, relapse rates have been reported between 30-60% (31, 33). It has been well documented that EBV induces PD-L1 expression in EBV-associated tumor cells while the TME of PCNSL has been shown to significantly express PD-1 and PD-L1, especially by copy gain and chromosomal translocation of chromosome 9p24.1 (34, 35). Furthermore, PCNSL, has been shown to harbor a higher Tumor Mutational Burden (TMB), which is associated with better response to anti-PD-1 therapies in solid tumors (34, 36). This data serves as the supporting basis for trials that utilize ICI therapy in PCNSL.

Current data on the efficacy of ICI therapy in PCNSL is mainly based on a small number of case series. Nayak et al. report 5 of 5 patients (4 with r/r PCNSL and 1 with CNS relapse of Primary Testicular Lymphoma [PTL]) treated with Nivolumab having a clinical response, 3 of which had PFS between 13-17 months (37). A more recent case series reported by Gavrilenko et al. also reported activity of Nivolumab in 8 patients with PCNSL and 1 with CNS relapsed PTL in which ORR was 78%, CR rate was 33.3%, with a 2-year OS of 44% and median OS of 12 months. The 2-year PFS was 26% with a median PFS of 12 months (38). Pembrolizumab has been reported to induce prolonged remission in at least 3 of 5 patients with r/r PCNSL (39). Finally, ICI in combination with Rituximab induced a CR in 3 of 6 patients with either PCNSL or Secondary CNS Lymphoma (40).

Thus, it seems that ICI may have significant activity in the treatment of r/r disease, but no formal prospective studies have been published to date and more studies are needed. Fortunately, there are a number of active trials underway assessing ICI as monotherapy and in combination for r/r PCNSL, as shown in **Table 4**.

**Table 4:** Current clinical trials evaluating CPI in PCNSL.

NCT Number	Phase	Disease Focus	Interventions
NCT04462328	I	PCNSL, SCNSL	Durvalumab + Acalabrutinib
NCT04688151	I	R/R PCNSL	Durvalumab + Rituximab + Acalibrutinib
NCT04022980	I	New PCNSL >65y	Nivolumab Consolidation
NCT03798314	I	R/R PCNSL	Nivolumab + Pomalidomide
NCT04609046	I	New PCNSL	Nivo + Lenalidomide + Rituximab + Methotrexate
NCT04421560	I/II	R/R PCNSL	Pembro + Rituximab + Ibrutinib
NCT02779101	II	R/R PCNSL	Pembrolizumab
NCT03255018	II	R/R PCNSL	Pembrolizumab
NCT03212807	II	R/R EBV+ NHL, PCNSL, PTL	Durvalumab + Lenalidomide
NCT04401774	II	New PCNSL with persistent CSF circulating tumor DNA	Nivo Maintenance after first-line therapy
NCT03770416	II	R/R PCNSL	Nivolumab + Ibrutinib
NCT02857426	II	R/R PCNSL, R/R PTL	Nivolumab
NCT05425654	II	New PCNSL	Nivo Maintenance after RL-MPV

PCNSL Primary Central Nervous System Lymphoma, SCNSL Secondary CNS Lymphoma, R/R Relapsed or Refractory, Nivo Nivolumab, Pembro Pembrolizumab, EBV Epstein-Barr Virus, NHL Non-Hodgkin Lymphoma, PTL Primary Testicular Lymphoma, CSF Cerebrospinal Fluid, RL-MPV Rituximab + Lenalidomide + Methotrexate + Procarbazine + Vincristine.

## T-cell lymphomas

Since our understanding of the underlying pathology of T-cell NHL is limited, current therapy is largely based on treatments for B-cell NHL. This includes regimens such as cyclophosphamide, doxorubicin, vincristine, and prednisone, with or without etoposide (CHOP and CHOEP, respectively). Other forms of immunotherapies have shown improved outcomes, as seen in the ECHELON-2 Trial, in which combining Brentuximab vedotin with the standard chemotherapy, cyclophosphamide, doxorubicin, prednisone (BV-CHP) had an improved 5-year PFS and 5-year OS as compared to CHOP (41). Thus, it seems that immunotherapies, including ICI, have a role in treating T-cell NHLs. Significant PD-1 expression has been reported in the TME of many subtypes of PTCL such as Angioimmunoblastic T-Cell Lymphoma (AITL), PTLD-NOS, and ALK^-^ Anaplastic Large Cell Lymphoma (ALCL) (42, 43). Extranodal NK/T-cell Lymphoma (ENKTL), in contrast, does not seem to overexpress PD-1, though PD-L1 overexpression has been well documented in both the malignant cells and stromal cells. Still, the prognostic implications of PD-L1 expression remains mixed; though higher PD-1/PD-L1 and CTLA-4 expressing TMEs as being associated with worse prognosis (44). Further analyses suggest an inability of HCT to overcome these survival outcomes (45). Thus the prognostic implications of immune checkpoint molecules remains a significant knowledge gap that requires further investigation.

In assessing ICI activity in r/r PTCL, investigators unexpectedly saw a subset of patients experience disease hyper-progression, defined as progression within 2 months of treatment initiation, resulting in halting of clinical studies. Small studies have reported rates of hyper-progression as high as 50% while other have been terminated after interim analysis showed futility (46–48). Thus far, studies evaluating anti-PD-1-based therapy in r/r PTCL (as either monotherapy or in combination) have reported ORRs ranging from 36.4-50% and CRs from 0-35.7% (49–51). Iyer et al. reported an ORR ranging from 44-50% with just 2 patients experiencing hyper-progression with the combination of Pembrolizumab and the Histone Deacetylase Inhibitor (HDACi), Romidepsin (51). Thus, current data remains weak toward the utility for ICIs in r/r PTCL and the phenomenon of hyper-progression is not well understood. Further investigation is needed to understand the mechanisms driving hyper-progression and the optimal use of ICI-based therapy in r/r PTCL.

In contrast, ENKTL appears to have some benefit with ICI. The Orient-4 trial reported an ORR of 75% in patients treated with Sintilimab who failed a SOC asparaginase-containing regimen. The 24-month OS rate was 78% and median OS was not reached after the 30.4-month follow-up. A phenomenon of “pseudo-progression” was seen in 5 patients initially thought to have progressive disease and eventually showed a later response during the trial (52). Other studies have reported lower ORRs ranging from 38-40% and CRs 24-31% in r/r ENKTLs who have failed SOC therapies and were specifically treated with anti-PD-L1 therapies (53, 54). These findings are lower than expected given the fact that ENKTL tumors tend to have preferential PD-L1 expression in the TME as previously noted. Still, these data seem promising for r/r disease in which median OS without treatment is reportedly less than 6 months. Further investigation is needed to optimize the response rates of r/r ENKTL to ICI-based therapies. Other case reports have mentioned CRs in a small number of patients with combinations such as anti-PD-1 with a HDAC inhibitor (Chidamide), Etoposide, and Thalidomide, as published by Du et al. (55)

With responses to ICI in NK-/T-Cell Lymphomas (NKTCL) being mixed, predictors of response centered around PD-L1 expression have been well documented. Lim et al. identified a strong predictor of treatment response in patients who harbored a somatic mutation causing a structural rearrangement leading to a mutated PD-L1 (PD-L1^MUT^). When treated with ICI, these patients had higher ORRs and continued having a durable response as of publication (31-49 months) (56). However, additional studies have highlighted that relapsed ENKTL patients show higher frequencies of other mutations in JAK-STAT, NF-Kappa B, and PI3K-AKT pathways (57). These findings not only suggest mechanism of resistance, but also support the reasoning for ICI use in combination with other agents. Thus, further investigation is needed to evaluate effective ICI-based combination therapies with agents that target these other pathways. **Table 5** summarizes currently active studies evaluating ICI in several combination regimens.

**Table 5:** Current clinical trial evaluating CPI in T-Cell Malignancies.

NCT Number	Phase	Disease Focus	Intervention
NCT03240211	I	PTCL	Pembro + Decitabine + Pralatrexate
NCT04414969	I	ENKTL	Anti-PD-1 + PEG-Asparaginase + Chidamide
NCT03905135	I	R/R T-NHL	Avelumab + IL-15
NCT03161223	I/II	PTCL	Durvalumab + Paraltrexate, Romidepsin, or AZA
NCT03278782	I/II	PTCL	Pembro + Romidepsin
NCT03598998	I/II	PTCL	Pembro + Pralatrexate
NCT04795869	II	R/R PTCL	Pembro + Brentuximab vedotin
NCT03703050 (NIVO-ALCL)	II	R/R ALK^+^ ALCL	Nivolumab

PTCL Peripheral T-Cell Lymphomas, Pembro Pembrolizumab, ENKTL Extranodal Natural Killer Cell/T-cell Lymphoma R/R Relapsed or Refractory, T-NHL T-cell Non-Hodgkin Lymphoma, ALCL Anaplastic Large Cell Lymphoma.

## Multiple myeloma

The current treatment paradigm for Multiple Myeloma (MM) involves triple combination regimens with an immunomodulatory drug (IMiD) such as Lenalidomide, a proteosome inhibitor such as Bortezomib, and a steroid followed by autoHCT. Despite a bevy of treatment options for MM, most patients will become refractory to available treatments after periods of remission and eventually succumb to their disease (58).

Circulating T-cells from r/r MM patients are known to have significantly abundant PD-1 and LAG3 expression, in which higher expression levels are predictive of poorer patient survival, suggesting a role for targeting these checkpoint molecules (59, 60). Multiple Phase I/II studies investigating Pembrolizumab in combination with an IMiD and steroid yielded ORRs ranging from 50-60% in refractory patients (61, 62). Results of these early studies spawned Phase III investigations in newly diagnosed (KEYNOTE-185) or r/r (KEYNOTE-183, CheckMate 602) MM populations. Unfortunately, these studies were prematurely halted after interim data suggested that Pembrolizumab-containing regimens had higher rates of treatment related deaths (4 vs 0) and higher rates of grade 3/4 Adverse Events (AEs) (63–65). Interestingly, KEYNOTE-183 noted that at a median follow up of 8.1 months, ORRs were similar between anti-PD-1 plus SOC and SOC alone at 62% vs 64%, respectively. Post-hoc analysis suggests that patients randomized to the Pembrolizumab group had a greater proportion of high-risk features such as stage 3 disease, high-risk cytogenetics, and extramedullary disease (65). Additionally, the previously mentioned study by Armand et al. saw a 0% ORR in a heavily pretreated MM population treated with combination Nivo/Ipi or Nivo/Liri, though the study was not powered for efficacy (12). Still, these current data provide a cautionary tale for future studies and emphasize the need to better characterize the TME of MM in order to identify those who would respond to ICI-based therapies.

Reasons for the lack of positive results remains unknown, but it could be speculated that specific polymorphisms within the PD-1 and CTLA-4 genes may affect response to ICI therapy. Gonzalez-Montes et al. identified a CTLA-4 polymorphism (CTLA4 rs231775 AA/AG) that served as an independent predictor of progressive disease (66). They reported that the AA/AG genotype was associated with median PFS of 32 months vs 96 months for the normal GG genotype while 5-year survival rate was half (25% vs 55%, respectively). Studies such as these highlight a need to find sub-populations of MM patients appropriate for ICI-based therapies. A review of active studies is outlined in **Table 6** and highlights several trials evaluating ICI therapy both in the upfront and relapsed/refractory setting.

**Table 6:** Current clinical trials evaluating CPI in MM.

NCT Number	Phase	Disease Focus	Intervention
NCT02616640	I	MM	Durvalumab +/- Pomalidomide
NCT05338775 (TRIMM-3)	I	R/R MM	Anti-PD-1 with Talquetamab or Teclistamab
NCT03267888	I	R/R MM	Pembro + RT
NCT02603887	I	sMM	Pembro
NCT03605719	I	R/R MM	Nivo + Carfilzomib + Dexamethasone + Pelareorep
NCT02681302 (CPIT001)	I/II	MM	Ipi or Nivo after AutoHCT
NCT03292263	I/II	MM	Nivo with AutoHCT
NCT01592370	I/II	MM, NHL, HL	Nivo + Daratumumab
NCT03848845 (DREAMM 4)	I/II	R/R MM	Pembro + GSK2857916
NCT05514990 (AMBUSH)	I/II	R/R MM	Pembro + Bortezomib +/- Pelareorep
NCT02906332	II	hrMM	Pembro + Lenalidomide after AutoHCT
NCT05191472	II	R/R MM	Pembro after CAR-T
NCT05204160	II	MM	Pembro after CAR-T
NCT04119336	II	R/R MM	Nivo + Ixazomib + Cyclophosphamide + Dexamethasone
NCT03184194	II	MM	Nivo + Daratumumab +/- Cyclophosphamide

MM Multiple Myeloma, R/R Relapsed/Refractory, Pembro Pembrolizumab, RT Radiation Therapy, sMM Smoldering MM, Nivo Nivolumab, Ipi Ipilimumab, AutoHCT Autologous Hematopoietic Stem Cell Transplant, hrMM High Risk MM, CAR-T Chimeric Antigen Receptor T-Cell.

GSK2857916 (anti-BCMA antibody).

## MDS/AML

The current treatment paradigm for AML is with 7 + 3 induction consisting of an anthracycline and Cytarabine with or without Gemtuzumab ozogamicin (an anti-CD33 antibody-drug conjugate) followed by consolidative chemotherapy as a bridge to allogeneic HCT (alloHCT). It is estimated that 50% of patients will relapse after alloHCT for which several targeted immunochemotherapeutic regimens have been approved, however most patients will ultimately relapse and succumb to their disease (34).

Like other malignancies, cell surface checkpoint molecules have been identified in acute leukemia patients with some markers being predictive of outcomes. PD-L1 positivity has been documented in AML, both at initial diagnosis and in relapse (67). Furthermore, PD-L1 expression has exclusively correlated with worse outcomes in AML separate from other prognostic factors like blast count, immunophenotype, and cytogenetic mutations (68). Hypomethylating agents (HMA), commonly used in the r/r setting, are thought to induce PD-1/PD-L1 expression (69). Based on this premise, several studies have investigated the combination of HMA and PD-1/PD-L1 agents. Initial studies of r/r AML patients treated with combination PD-1/PD-L1 with HMA yielded ORRs ranging from 17-58% (70, 71). HMA-naïve and HMA-pretreated AML patients treated with Azacitidine (AZA) and Nivolumab had reported ORRs of 58% and 22%, respectively. Responders to AZA and Nivolumab had higher CD3^+^, CD4^+^ T_eff_, and CD8^+^ T cells in their pretherapy bone marrow aspirates compared with non-responders. Those same non-responders had significantly higher CTLA-4 upregulation on CD4^+^ effector T-cells after nivolumab dosing (70). This would suggest that selective T-lymphocyte depletion in advanced salvage patients plays a role in response to ICI-based therapy. It is possible that multiple rounds of chemoimmunotherapy selects for exhausted effector T-cells and/or selects for alternative inhibitory pathways. Other studies evaluating AZA with Nivolumab were terminated early when it was determined that the combination arm had significantly more early patient death and no difference in efficacy when compared to AZA alone (72). The authors attributed the higher death rate to the inclusion of patients with an ECOG of 2 or more, unrecognized autoimmune complications, and patients with FLT3-ITD mutations. Thus, it appears that ICI therapies may play a limited role in AML with known driver mutations and further investigation is needed to assess these mechanisms of resistance.

In the treatment of Myelodysplastic Syndrome (MDS), response to PD-1/PD-L1 signal blockade has been mixed. A Phase II study of AZA with Durvalumab vs AZA alone as first-line for High-Risk MDS patients found that although ORR was numerically greater in the combination group (61% vs 47%, P=0.18) it was not statistically significant. In contrast, OS was numerically lower, though also not statistically significant (11.6 vs 16.7 months, P=0.74). In phenotyping the tumor samples, AZA exposure did increase PD-L1 expression on monocytes and granulocytes, but not tumor blasts (72, 73). Garcia et al. report an ORR of 52% in pre-transplant patients treated with Ipilimumab and Decitabine, though responses were short lived (74). Decitabine was thought to act directly on the leukemic cells while Ipilimumab was thought to act on TILs, and was thus dependent on the immunophenotype of the infiltrating lymphocytes (74). These data suggest that ICI-therapy can potentially have a role in the treatment of MDS/AML, but further studies are needed to better understand the effects of a varying immunophenotype of TILs on response rates to ICI. Despite these mixed data, novel checkpoint targets are showing promising results in treating MDS/AML which are addressed in following sections.

Many studies aim to further assess the utility of ICI in all lines of therapy, as displayed in **Table 7**. Notably, the BLAST MRD AML-1 and -2 trials aim to incorporate Pembrolizumab to SOC therapies for both fit and unfit newly diagnosed AML patients. Nivolumab is being assessed by two groups as maintenance therapy to lengthen remission duration in AML patients. Also interestingly, the utility of Nivolumab is being evaluated in the treatment of TP53^+^ AML. As more data is reported from these studies, ICI therapy may very well have a role in treating myeloid malignancies.

**Table 7:** Current clinical trials evaluating CPI in MDS/AML.

NCT Number	Phase	Disease Focus	Intervention
NCT02846376	I	MDS, AML	Nivo alone vs Ipi alone vs Nivo/Ipi after alloHCT
NCT03600155	I	HR-MDS, R/R AML	Nivo/Ipi after HCT
NCT04277442	I	TP53^+^ AML	Nivo + Decitabine + Venetoclax
NCT01757639	I	R/R HR-MDS, R/R AML	Ipilimumab
NCT02890329	I	R/R MDS, R/R AML	Ipilimumab + Decitabine
NCT02936752	I	MDS after HMA	Pembro + Entinostat
NCT03969446	I	New and R/R MDS/AML	Pembro + Decitabine +/- Venetoclax
NCT02117219	I	MDS after HMA	MEDI4736 alone vs MEDI4736 + Tremelimumab with or without AZA
NCT03144245	I	R/R AML	AMV564 +/- Pembro
NCT02464657	I/II	HR-MDS, AML	Nivo + 7 + 3 Induction
NCT02996474	I/II	R/R AML	Pembro + Decitabine
NCT02935361	I/II	R/R MDS, R/R CMML	Atezolizumab + Guadecitabine
NCT03417154	II	HR-MDS, R?R AML	Nivo + low dose Cyclophosphamide
NCT02530463	II	MDS	Nivo and/or Ipi +/- AZA
NCT02397720	II	New AML, R/R AML	Nivo + AZA vs Nivo/Ipi + AZA
NCT04913922	II	R/R AML, elderly AML	Nivo + Relatlimab + AZA
NCT02532231	II	AML in CR	Nivo maintenance after CR
NCT02275533 (REMAIN)	II	AML in CR	Nivo maintenance after CR
NCT02775903	II	HR-MDS, elderly AML	Durvalumab + AZA vs AZA alone
NCT03769532	II	R/R NPM1+ AML	Pembro + AZA
NCT02845297	II	R/R AML, elderly AML	Pembro + AZA
NCT02708641	II	elderly AML	Pembro maintenance after CR
NCT02771197	II	Non-favorable risk AML	Pembro following AutoHCT with Flu/Mel lymphodepletion
NCT03094637	II	MDS	Pembro + AZA
NCT02768792	II	R/R AML	Pembro + HiDAC
NCT04214249 (BLAST MRD AML-1)	II	New AML	7 + 3 +/- Pembro
NCT04284787 (BLAST MRD AML-2)	II	New Unfit AML	AZA + Venetoclax +/- Pembro
NCT03092674	II/III	HR-MDS, elderly AML	Nivo + AZA vs Midostaurin + AZA vs AZA alone vs Decitabine + Cytarabine

Nivo Nivolumab, Ipi Ipilimumab, hr-MDS High-Risk MDS, R/R Relapsed or Refractory, AZA Azacitidine, Pembro Pembrolizumab, HMA Hypomethylating Agent, 7 + 3 Cytarabine + Anthracylcine (i.e. Idarubicin or Daunorubicin), CMML Chronic Myelomonocytic Leukemia, CR Complete Remission, Flu/Mel Fludarabine-Melphalan, HiDAC High-Dose Cytarabine.

MEDI4736 (anti-PD-L1 antibody). AMV564 (bispecific anti-CD33/CD3 antibody).

## ALL

SOC therapy for Acute Lymphoblastic Leukemia (ALL) is centered around consolidative chemoimmunotherapy combinations, which include Blinatumomab (an anti-CD20 monoclonal antibody) and Inotuzumab ozogamicin (an anti-CD22 antibody-drug conjugate). Chemoimmunotherapy traditionally serves as a bridge to HCT. Lately, studies characterizing the TME of ALL suggest a potential role for ICI therapy. It had been shown that relapsed TMEs had higher PD-1 expression and intensity on both CD4^+^ and CD8^+^ T-cells than TMEs that were either with persistent disease or in CR. Interestingly, despite patients who were newly diagnosed with ALL having lower overall PD-1 expression, the signal intensity on individual CD4^+^ or CD8^+^ T-cells was no different than patients with relapsed disease. There was also no difference in PD-L1 expression on the leukemic blasts when based on disease status (75). This suggests that an increase in absolute PD-1^+^ effector T-cells is a major driver of immune evasion and serves as a viable therapeutic target.

To assess efficacy of ICI in the treatment of ALL, Cassaday et al. investigated the ability of Pembrolizumab to convert patients with positive minimal residual disease (MRD) to CR with negative MRD. The trial was halted early due to only 1 patient achieving MRD negativity after the first 200mg cycle of Pembrolizumab (76). Other studies have documented the importance of CD4^+^ T-cell exhaustion in predicting outcomes for ALL patients. Tracy et al., showed that in mouse models with B-ALL, OS was better in mice treated with nilotinib plus anti-PD-L1 blockade compared to nilotinib alone. They report that anti-PD-L1 blockade lead to clonal expansion of leukemia-specific CD4^+^ Helper and Cytotoxic T-cells while reducing markers of T-cell exhaustion (77). This suggests potential activity in combination Tyrosine Kinase Inhibition (TKI) with ICI in Ph+ B-ALL, which was being further assessed in a Phase I study (NCT02819804), but the trial has been terminated due to funding and accrual issues. These data suggest that T-cell exhaustion plays an important role in response to ICI-therapy with ALL and is a significant area in need for further investigation to better understand the pathophysiology and mechanisms of resistance with ALL.

There are a small number of studies currently assessing ICI activity in ALL. NCT02879695 is a Phase I study assessing combination Blinatumomab plus Nivolumab with or without Ipilimumab in poor-risk CD19^+^ r/r B-ALL patients. Similarly, both NCT03160079 and NCT03512405 are Phase I/II studies looking at the safety and efficacy of combination Blinatumomab with Pembrolizumab in r/r B-ALL.

## ICI with CAR-T therapies

Chimeric Antigen Receptor T Cells (CAR-T) are effector T-cells with modified T-Cell Receptors (TCR) targeting specific cell-surface antigens and are engineered to be activated independently of MHC. Thus far, there are six manufactured CAR-T products FDA approved to treat ALL, NHL (DLBCL, FL, Mantle Cell Lymphoma), and MM (78, 79). PD-1 expression on the infused CAR-T cells has been, in part, thought to be a determining factor influencing rates of response. Studies have shown PD-1/PD-L1 blockade restored CAR-T effector function, thus suggesting this interaction may cause T-cell exhaustion within the TME (80). Porter et al. reports that patients with lower expression of CD8+PD-1+ T-cells had a better response to CAR-T, suggesting that the inhibitory effect of PD-1 signaling plays a role in controlling the anti-tumor effect of CAR-T therapy (81). Thus, ICI in combination with CAR-T has been of great interest.

The ZUMA-6 trial is currently evaluating the safety and efficacy of Axicabtagene Ciloleucel in combination with Atezolizumab for r/r DLBCL (NCT02926833). The end of Phase I analysis reported that out of 12 patients, the ORR was 90% with 6 patients achieving CR and 3 achieving PR. Using the ZUMA-1 data for comparison, CAR-T cell expansion was over 2-fold higher in ZUMA-6 (82). These results have allowed for the opening of Phase II of the ZUMA-6 trial. Additionally, Hirayama et al. reported preliminary data evaluating combination of a CD-19-specific 4-1BB-costimulated CAR-T (JCAR014) with Durvalumab in treating r/r DLBCL. Of the 12 patients evaluable, ORR was 50% (5 with CR and 1 with PR). CAR-T expansion was seen in the peripheral blood within 14 days of infusion with higher peaks observed in responding patients (83). These data serve as proof of concept to a synergistic effect of ICI with CAR-T therapy resulting in a more robust effector T-cell expansion, which in turn leads to improved outcomes and more durable responses, thus providing an exciting area for further investigation.

There has been additional interest in the utility of ICI in r/r disease after CAR-T therapy. Chong et al. evaluated the efficacy of Pembrolizumab for r/r NHL after CD-19-specific 4-1BB-costimulated CAR-T, but the best ORR seen was 25% (84). Separately, Li et al. evaluated the use of Pembrolizumab or Nivolumab with a second CAR-T infusion in pediatric patients with r/r ALL or B-lymphoblastic lymphoma early after a first CAR-T infusion. Of 14 patients enrolled, 3 patients established B-cell aplasia, 2 of which had ongoing aplasia with Pembrolizumab maintenance therapy. Of 4 patients with bulky extramedullary disease, 2 achieved CR and 2 had PR (85). These preliminary data suggest that at least a subset of r/r patients to CAR-T could benefit from subsequent ICI by inducing a re-expansion of the CAR-T population. But this appears to only be most effective when the primary mechanism of tumor persistence is checkpoint inhibitor-mediated T-cell exhaustion. As Deng et al. showed, higher PD-1 expression was associated with poorer response rates and PD-1 was only expressed in a small subset of cells. Additionally, other exhaustive markers such as LAG-3 and TIM-3 had stronger correlation to a lower response rate (86). Thus far, data seems to be more promising for ICI used adjunctively with CAR-T therapy rather than as a subsequent line of therapy, but alternative checkpoint molecules (discussed later in this review) may serve an important role in mechanisms of resistance. Further studies are needed to understand the best utilization of ICI with CAR-T and how to best target alternative checkpoint molecules. **Table 8** summarizes active studies evaluating ICI given both concurrently and after CAR-T therapy in several hematologic malignancies.

**Table 8:** Current clinical trials evaluating CPI with CAR-T therapy.

NCT Number	Phase	Disease Focus	Intervention
NCT05352828 (ACTION)	I	R/R cHL	Nivo + CAR-T
NCT04134325 (LCCC1852-ATL)	I	R/R cHL	Anti-PD-1 after CAR-T
NCT05191472	II	R/R MM	Pembro after CAR-T
NCT05204160	II	R/R MM	Pembro after CAR-T
NCT05385263	II	Stable/Progressed DLBCL	Nivo + CAR-T
NCT04205409	II	R/R CLL R/R NHL R/R MM	Nivo after CAR-T
NCT05672173	II	RT-DLBCL	Nivo + Ibrutinib + CAR-T

R/R Relapsed/Refractory, cHL Classical Hodgkin Lymphoma, Nivo Nivolumab, CAR-T Chimerica Antigen Receptor T-cell, MM Multiple Myeloma, Pembro Pembrolizumab, DLBCL Diffuse Large B-Cell Lymphoma, Nivo Nivolumab, CLL Chronic Lymphocytic Leukemia, NHL Non-Hodgkin Lymphoma, RT-DLBCL Richter Transformation-DLBCL.

## ICI with allogeneic stem cell transplant

In the context of allogeneic stem cell transplant (alloHCT), effective response requires a delicate balance between allowing a graft-vs-leukemia effect and suppressing graft-vs-host disease (GVHD). Therefore, there is concern that the use of ICI therapy either before or after alloHCT would increase the risk and severity of GVHD. As Nguyen et al. reported in their worldwide literature review, rates of ICI-induced GVHD were found to be 57% with Nivolumab, 24.7% with Pembrolizumab, and 12.9% with Ipilimumab. Mortality rate was 25.8%. The majority of the cases reviewed were of ICI therapy after alloHCT (87).

But, despite the concern for ICI-induced GVHD, early data suggest benefit to ICI after alloHCT. Early studies evaluating ICI after alloHCT for r/r HL report ORRs ranging from 75-95%, 1-year PFS from 47-58%, and 1-year OS from 78-89%. Rates of both Acute and Chronic GVHD ranged from 15-30% (88, 89). Other early studies looking at ICI after alloHCT for r/r AML report lower ORR rates ranging from 20-32%, 1-year PFS ranging from 18.2-23%, and 1-year OS around 56% (74,) (90–92). It is speculated that the difference in responses is secondary to downregulation of HLA-1 complexes resulting in decreased antigen presentation to CD8+ TILs. It is further speculated that an increase in Tregs following ICI is a compensatory mechanism of resistance in AML (74). Thus, preliminary data would suggest activity with ICI after alloHCT at least in the treatment of HL, but further studies are needed to assess benefits in other hematologic malignancies and whether this outweighs the risk of GVHD.

In contrast, several studies, have evaluated the safety and efficacy of ICI therapy before alloHCT, mainly for r/r HL and NHL. Rates of acute GVHD ranged from 33-44% while rates of chronic GVHD (with patients receiving varying GVHD prophylaxis) ranged from 35-41%. The 1-2 year OS ranged from 77-89% and 1-2 year PFS ranged from 74-76%, thus suggesting still a significant survival benefit (93, 94). Further studies have shown improvement in controlling GVHD in these patients with posttransplant cyclophosphamide (PTCy) as prophylaxis. Ikegawa et al. showed that PTCy could successfully restore T-cell homeostasis and ameliorate clinical GVHD in mouse models (95). Additionally, Oran et al. showed that AML/MDS patients who had prior ICI therapy and later underwent alloHCT had lower rates of acute GVHD when treated with PTCy prophylaxis as compared to those who were not. Rates of acute GVHD in these patients receiving PTCy were similar to patients who underwent alloHCT without prior ICI therapy (96). Tschernia et al. further showed no difference in 1-year OS, 100-day mortality, or rates of grade 3-4 GVHD in AML patients who received high-dose Cytarabine with Pembrolizumab followed by alloHCT to a historical control group who received SOC without ICI. These patients all received PTCy as part of their GVHD prophylactic regimen (97). Thus, these data support the safety of ICI therapy prior to alloHCT and suggest that PTCy may negate the inherent increased risk of both acute and chronic GVHD observed with pre-transplant ICI therapy. Even in the setting of haploidentical transplantation with PTCy, reports show no difference on OS, nor a significant increase in 100-day incidence of grade 2-4 acute GVHD or chronic GVDH. Data also suggests a 2-year relapse incidence of 0% with prior ICI vs 20% without prior ICI (98). Overall, these data support the idea that ICI therapy prior to HCT incurs a higher risk of both acute and chronic GVHD, but the addition of PTCy likely mitigates this increased risk and thus makes HCT after ICI therapy a viable and effective treatment option of r/r hematologic malignancies. Whether there is an overall survival benefit or improved response rate remains to be answered. Though most trials have evaluated ICI after alloHCT in the context of HL, more studies have evaluated this strategy in other hematologic malignancies as well, as outlined in **Table 9**.

**Table 9:** Published trials evaluating CPI before and after AlloHCT.

Name/Code	Year	Phase	Disease/Status	Sample Size	Intervention	Response/AEs	Duration	Ref #
PMID: 28270452	2017	Retrospective	R/R HL	20	Nivo after AlloHCT	ORR: 95% OS: 78.7% GVHD rate: 30%	1-yr PFS: 58.2%	(88)
PMID: 32748216	2020	Retrospective	R/R HL	25	Anti-PD-1 before AlloHCT	aGVHD: 47.1% aGVHD with PTCy: 14.6% 1-yr OS: 81.3%		(89)
PMID: 32748216	2020	Retrospective	R/R HL	20	Anti-PD-1 after AlloHCT	ORR: 75% CR: 40% aGVHD: 15% cGVHD: 30% 1-yr OS: 89.7%		(89)
NCT01822509	2016	I	R/R AML R/R HL R/R NHL R/R MDS R/R MM R/R ALL	28	Ipi after AlloHCT	ORR: 32% 1-yr OS: 49% GVHD: 14%		(90)
NCT02981914	2023	I	R/R AML R/R MDS R/R cHL R/R DLBCL	12	Pembro after AlloHCT	ORR: 22% irAE (any grade): 42% grade 3-4 irAE: 25% No GVHD observed		(92)
NCT02890329	2023	I	New and R/R MDS New and R/R AML	23	Ipi + Decitabine Pre-HCT	ORR: 52% irAE rate: 48%	mDOR: 6.14 mo	(74)
NCT02890329	2023	I	New and R/R MDS New and R/R AML	25	Ipi + Decitabine Post-HCT	ORR: 20% irAE rate: 44%	mDOR: 4.46 mo	(74)

R/R Relapsed/Refractory, cHL Classical Hodgkin Lymphoma, Nivo Nivolumab, AlloHCT Allogeneic Hematopoietic Stem Cell Transplant, ORR Objective Response Rate, CR Complete Response, OS Overall Survival, aGVHD Acute Graft-Vs-Host Disease, cGVHD Chronic GVHD, PFS Progression Free Survival, PTCy Posttransplant Cyclophosphamide, AML Acute Myeloid Leukemia, NHL Non-Hodgkin Lymphoma, MDS Myelodysplastic Syndrome, MM Multiple Myeloma, ALL Acute Lymphoblastic Leukemia, DLBCL Diffuse Large B-Cell Lymphoma, Ipi Ipilimumab, Pembro Pembrolizumab, irAE Immune-Related Adverse Event, mDOR Median Duration of Response.

As such, both ICI and alloHCT are effective treatment approaches for several hematologic malignancies with a large portion of data supporting their use in r/r HL. The optimal timing of these therapies remains to be established, but it seems the risk of GVHD after ICI therapy is at least mitigated with the use of PTCy. Thus, more studies are needed to better understand where ICI fits best in relation to alloHCT. A review of active trials shows numerous studies focusing within this field. There are several Phase I trials evaluating ICI in combination or as monotherapy after HCT for myeloid and lymphoid malignancies (NCT02846376, NCT01822509, NCT00060372, NCT03600155, NCT03146468-NIVALLO, NCT04361058). NCT01919619 is a trial reevaluating the use of Lenalidomide and Ipilimumab after HCT. NCT02981914 is a pilot study evaluating Pembrolizumab after alloHCT. NCT04128020 is a Phase I trial evaluating the combination of Azacitadine with Nivolumab following reduced-intensity alloHCT for AML and high-risk MDS. NCT04635735 is a Phase I/II trial evaluating the safety and efficacy of Ipilimumab after HCT for r/r Multiple Myeloma.

## Other targetable checkpoint molecules

### LAG-3

Lymphocyte-activation gene 3 (LAG-3) is a transmembrane protein expressed on T-lymphocytes and NK cells. Binding of LAG-3 to MHC-II results in suppression of autoimmune and anti-cancer immunity (99). Additionally, the binding of LAG-3 to the soluble liver-secreted Fibrinogen-Like protein 1 (FGL-1) has been shown to inhibit CD8^+^ T cell-mediated anti-tumor effects, but the significance of this interaction in hematologic malignancies remains to be elucidated (100). More recently, increased LAG-3 expression has been correlated to treatment resistance in a number of hematologic malignancies including CLL, FL, and DLBCL (101–104). Relatlimab (BMS-986016, Bristol-Meyers Squibb) is a human IgG_4_ anti-LAG-3 monoclonal antibody (mAb). *In vitro* studies have shown that exposure of Relatlimab to peripheral blood mononuclear cells of CLL patients induced anti-leukemic activity and increased cytokine production (105). Further industry-sponsored trials have evaluated safety, tolerability, and efficacy of Relatlimab monotherapy or in combination with Nivolumab in r/r B-cell malignancies (NCT02061761). Preliminary data shows ORRs to Relatlimab and Nivolumab of 61.9% for ICI-naïve HL patients, 6.7% for DLBCL patients, and 15.0% for HL patients with prior ICI therapy. Durations of responses were 14 months, 1 month, and 6.37 months, respectively (106). Thus, based on this preliminary data, LAG-3 appears to be a potentially effective alternative target of checkpoint inhibition for at least some treatment resistant hematologic malignancies, but further investigation is needed to optimize its use.

Currently active trials include NCT03489369, a Phase I, open-label trial assessing safety, tolerability, and antineoplastic activity of an Anti-LAG-3 mAb (Sym022) in advanced solid tumors and lymphomas. The same investigators are further assessing Anti-PD-1 therapy in combination with either Anti-LAG-3 or Anti-TIM-3 in both advanced solid tumors and lymphomas (NCT03311412). The combination of Relatlimab with Nivolumab and Azacitadine for the treatment of AML is being investigated in the trial NCT 04913922. HLX26 is another anti-LAG-3 mAb being investigated in solid tumors and lymphomas in NCT05078593.

### TIM-3

T-cell immunoglobulin and mucin domain 3 (TIM-3) is known to be co-expressed with PD-1 on effector T-cells and binds to many ligands, though most significantly to galectin-9. This interaction is known to negatively regulate CD8+ T-cell activation and Th1-type immunity by inducing cell death (107). TIM-3 appears to be a marker of exhaustion and an increased number of exhausted PD-1^+^/TIM-3^+^ CD8^+^ T-cells has been associated with disease progression and poorer prognosis in AML (108, 109). Preliminary *in vivo* studies have shown that blockade of TIM-3 prevents engraftment of leukemic stem cells without inhibiting normal stem cell engraftment (110). Furthermore, galectin-9 knockout mice are more resistant to AML morbidity/mortality while double blockade of PD-1/PD-L1 and TIM-3/galectin-9 as associated with reduced leukemic burden (111). TIM-3 expression has also been found to correlate to poor prognosis in other hematologic malignancies such as DLBCL, ALL, MDS, Chronic Myelogenous Leukemia (CML), and Chronic Myelomonocytic Leukemia (CMML) (112–115). Thus blockade of TIM-3/galectin-9 is a potentially effective target for further treatment in several hematologic malignancies.

Sabatolimab (MBG453) is an anti-TIM-3 mAb that has been shown to enhance immune-mediated killing of TIM-3^+^ leukemic cells *in vitro* and is currently under investigation in clinical trials (116). STIMULUS-MDS1 evaluated the safety and efficacy of Sabatolimab with HMA as first line treatment in intermediate, high-risk, and very-high-risk MDS patients who were not eligible for high intensity chemotherapy. The investigators reported a median PFS of 11.1 months for patients receiving Sabatolimab compared to 8.5 months for the placebo group. ORRs were 49.2% and 37.1% with CR rates of 23% vs 21%, respectively. Median OS was 19.0 mo vs 18.0 mo. None of the aforementioned comparisons were statistically significant, though trended in favor for sabatolimab with HMA, which the authors speculated may be due to a delayed-onset of benefit (117). Subsequently, STIMULUS-MDS2 is evaluating the safety and efficacy of combination Azacitadine and Sabatolimab in high-risk MDS and CMML as first-line treatment for patients who are not eligible for high intensity chemotherapy (115). The results of this current study are anticipated to provide strong evidence toward the benefits of anti-TIM-3 blockade.

Interestingly, other mechanisms of TIM-3 blockade are also being explored, as Wu et al. have identified the small molecule, SMI402, which inhibits substrate binding of TIM-3 and has been shown to increase activity of CD8^+^ T cells and NK cells *in vitro* (118). Studies are underway to assess its safety and efficacy.

TIM-3 remains a relatively newly discovered target for ICI and there are several active early phase studies (**Table 10**) evaluating its benefits in hematologic malignancies. NCT03489343 is investigation Sym023, an anti-TIM-3 mAb, as monotherapy for r/r solid tumors and lymphoma for which there was no further standard of care treatment available. Preliminary data in patients receiving the highest dose of Sym023 had a reported ORR of 66.7% in ≤ 16 weeks of treatment and a median Time To Progression of 5.36 months (119). The Keyplus-001study (NCT05357651) is evaluating a bispecific antibody to PD-1 and TIM-3 (LB1410) in the treatment of both advanced solid tumors and lymphoma. NCT05216835 is a similar Phase I trial evaluated AZD7789, a different anti-PD-1/anti-TIM-3 bispecific antibody, in the treatment of r/r HL. As previously mentioned, NCT03311412 is evaluating Anti-PD-1 therapy in combination with either Anti-LAG-3 or Anti-TIM-3 in both advanced solid tumors and lymphomas. Sabatolimab is further being studied in the post-alloHCT setting in AML patients who achieve CR with positive MRD as a preemptive treatment alone or in combination with Azacitadine to potentially enhance the graft-vs-leukemia effect (NCT04623216). NCT05367401, NCT03066648, NCT03940352, and NCT04150029 are investigating Sabatolimab in combination with other novel therapies for MDS and AML.

**Table 10:** Current clinical trials evaluating anti-TIM-3 therapy.

NCT Number	Phase	Disease Focus	Intervention
NCT05357651 (Keyplus-001)	I	Lymphoma	LB1410
NCT03489343	I	R/R Lymphoma	Sym023
NCT03066648	I	hrMDS, AML	Sabatolimab +/- Spartalizumab Sabatolimab or Spartalizumab + HMA
NCT03940352	I	hrMDS, AML	HDM201 + Sabatolimab or Venetoclax
NCT04810611	I	lrMDS	Sabatolimab
NCT05426798	I	R/R MDS, R/R AML	TQB2618 + HMA
NCT05400876	I/II	R/R Lymphoma	TQB2618 + Penpulimab
NCT04623216	I/II	AML	Sabatolimab +/- AZA after AlloHCT
NCT05216835	I/II	R/R cHL	AZD7789
NCT04150029 (STIMULUS-AML1)	II	Unfit AML	Sabatolimab + AZA + Venetoclax
NCT03946670 (STIMULUS-MDS1)	II	hrMDS	Sabatolimab + HMA
NCT04878432	II	hrMDS	Sabatolimab + HMA
NCT04266301 (STIMULUS-MDS2)	III	hrMDS, CMML-2	Sabatolimab +AZA

R/R Relapsed/Refractory, hrMDS High-Risk Myelodysplastic Syndrome, lrMDS Low-Risk MDS, AML Acute Myeloid Leukemia, HMA Hypomethylating Agent, cHL Classical Hodgkin Lymphoma, CMML Chronic Myelomonocytic Leukemia, AZA Azacitidine.

LB1410 (Bispecific anti-TIM-3/PD-1 antibody). Sym023 (Anti-TIM-3 antibody). HDM201 (TP-53-MDM2 small molecule inhibitor). TQB2618 (anti-TIM-3 antibody).

### TIGIT

T cell immunoreceptor with immunoglobulin and ITIM domain (TIGIT) is a checkpoint inhibitory protein expressed on effector T-cells, regulatory T-cells, and NK cells and primarily binds to CD155 and CD112 which are mainly expressed on dendritic cells, macrophages, and lymphocytes (120, 121). CD155 appears to be the primary ligand, in which binding results in downregulation of the T-cell Receptor while also increasing the production of anti-inflammatory cytokines such as IL-10 (122–126). TIGIT is known to be upregulated in TILs in HL and has been associated with poor prognosis (127). Advanced stage CLL patients have also been seen to have higher TIGIT^+^ T cells (128). Additionally, both r/r AML and MM patients have been shown to have higher levels of TIGIT^+^ CD8^+^ T-cells and have been associated with relapse after stem cell transplant (129, 130). More recently, TIGIT has been implicated as a major cause of relapse after CAR-T for Mantle Cell Lymphoma (131). Thus TIGIT appears to be an important mechanism of immune evasion for hematologic malignancies, but further understanding of its significance and whether it is an effective target for therapy remains unknown.

Tiragolumab is a new anti-TIGIT mAb and has shown promising anti-cancer activity in solid tumors. There are small number of active studies looking at the benefits of TIGIT blockade in hematologic malignancies. NCT04150965 is a Phase I/II trial evaluating anti-LAG-3 and anti-TIGIT as monotherapy followed by Pomalidomide and Dexamethasone for r/r MM after prior therapy. NCT05315713 is an open-label, Phase I/II trial evaluating combination Mosunetuzumab (anti-CD20/CD3 bispecific antibody) with Tiragolumab, with or without Atezolizumab for r/r FL and DLBCL. HLX301 is a bispecific antibody to PD-L1 and TIGIT being evaluated in a Phase I study for advanced solid tumors and lymphomas (NCT05390528), while HLX53 is an anti-TIGIT Fc fusion protein also being investigated in solid tumors and lymphomas.

### CD47

Cluster of differentiation 47 (CD47) is a transmembrane protein that is found on many cells, but most notably macrophages. It specifically binds to signal-regulatory protein α (SIRPα) expressed on myeloid cells which then inhibits macrophage-mediated phagocytosis and is known as a “don’t eat me signal” (132). CD47 upregulation has been reported in hematologic malignancies such as NHL, MM, and Leukemias and is associated with poorer prognosis (132–134). Blockade of the CD47-SIRPα interaction primarily leads to phagocytosis of cancer cells, but a significant AE is severe hemolysis due to RBCs expressing CD47. Blockade of this signaling also enhances antigen presentation as well as cross presentation on dendritic cells and leads to priming of CD8^+^ T-cells while also increasing neutrophil-mediated tumor cell death (135). Majeti et al. showed that *in vivo* blockade of the CD47-SIRPα interaction enabled phagocytosis of AML leukemic stem cells, reduced tumor burden, and prevented engraftment, thus laying the ground work for further clinical studies (136).

Magrolimab (Hu5F9-G4) is an anti-CD47 antibody that has shown a tolerable side effect profile in recent early phase trials, with the most common side effect being anemia. Advani et al. evaluated Magrolimab with Rituximab for r/r NHL and reported an ORR of 50% with CR of 36%. At a median follow-up of 8.1 months, 91% of responses were ongoing (137). Most notably, Magrolimab has been investigated in combination with Azacitadine in the treatment of high-risk MDS and AML patients as first-line therapy. The investigators saw an ORR in MDS of 91% and 64% in AML. Median duration of response was not reached as far out as 9.4 months. Remarkably, 6-month OS in MDS and TP-53 mutant AML were 100% and 91%, respectively (138). This promising data has led to further Phase III trials (ENHANCE trial) and provide an exciting potentially effective treatment option for a patient population with historically poor outcomes. Further ongoing trials are investigating Magrolimab in combination with Rituximab (NCT0352714), Acalbrutinib (NCT03527147), and Mogamulizumab (anti-CCR4, NCT03527147).

CD47 blockade is an exciting new therapeutic approach with drug developers finding several ways to target this molecule. CC-90002, another anti-CD47 mAb was investigated in combination with Rituximab for r/r NHL, but ORRs were merely 13% (139). It also did not seem to have any activity in r/r MDS or AML (140). No further phase II trials have been conducted thus far. Other additional anti-CD47 antibodies include IBI-88 (Letaplimab), AK117 (Ligufalimab), and TJ011133 (Lemzoparlimab) which are currently being investigated in combination therapies for the treatment of NHL, MDS, and AML. Lemzoparlimab interestingly has a unique binding epitope which spares RBC opsonization and therefore has been shown to have lower hematologic toxicity (141). When used in combination with Rituximab for r/r CD20+ NHL, early reports show an ORR of 57% (142). Other small studies have evaluated Lemzoparlimab monotherapy for r/r AML and found a tolerable AE profile with a small number of primary refractory patients achieving a morphologic leukemic-free state after two cycles (143). More studies are needed to best understand the utility of these novel agents.

Another approach to CD47 blockade is recombinant proteins using a SIRPα extracellular motif fused to a Fc fragment of the heavy chain of immunoglobulin. This approach has the particular advantage of having high affinity with a low molecular weight, suggesting better tumor infiltration by simple diffusion across vascular membranes (144). Both TTI-621 and TTI-622 are fusion proteins with a SIRPα motif link to an IgG1 Fc and IgG 4 Fc, respectively. Horwitz et al. conducted the First-In-Human Phase I trial with TTI-621 in r/r hematologic malignancies. Preliminary data shows that the most common AEs were infusion reactions (40-50%) and thrombocytopenia (30%). ORRs to single agent TTI-621thus far were found to be 20% for NHL (including CTCL, PTCL, and DLBCL) (145). Intralesional TTI-621 has also been found to be active in Mycosis Fungoides and Sezary Syndrome with a 90% response rate in the injected lesions. Interestingly, 80% of patients saw response in distal non-injected lesions as well suggesting systemic effects with intralesional injection (146). In contrast to TTI-621, TTI-622 does not bind to RBCs and is currently being studied as monotherapy for r/r NHLs as well. Preliminary data from Patel et al. reports an ORR of 33% for heavily treated r/r NHLs, which includes DLBCL, CTCL, PTCL, and FL (147). ALX148 (Evorpacept) is another high affinity SIRPα motif linked to an inactive IgG1 Fc. It is currently being studied in combination with Rituximab for r/r CD20^+^ NHL (ASPEN-01) in which preliminary data has reported a 14-month ORR of 40.9% and 9-month ORR of 63.6% (148). Lastly, IMM01 is a SIRPαIgG1 fusion protein with weak erythrocyte conjugation to lower the risk of hemolysis. It is currently being studied in r/r HL and NHL as monotherapy in which preliminary reported data shows an ORR of only14.3% (149). These data are a good starting point in assessing recombinant fusion proteins as a mode of CD47 blockade and could potentially allow for better tissue penetration and outcomes.

Finally, bispecific antibodies are a third approach to CD47 blockade while also combining a second immunotherapeutic target for synergistic effect. IMM0306 is a bispecific antibody to CD47 and CD20. It has shown *in vitro* effect with cancer killing ability while not binding to human RBCs. In mouse models for lymphoma, it had superior activity when paired with Lenalidomide as compared to other single agent therapy and combination Rituximab with Lenalidomide (150). TG-1801 is a bispecific antibody to CD47 and CD19. Similarly, preclinical studies have shown potent anticancer activity for numerous B-cell malignancies and has synergistic effect when used in combination with Rituximab (151).

CD47 is a promising and highly anticipated novel target for checkpoint-inhibitor blockade in which early trials (**Table 11**) are showing significant efficacy in anti-cancer activity. This becomes especially exciting in the treatment of TP-53 mutated AML for which treatment is difficult and response rates are poor. It is also exciting for treatment of other relapsed/refractory disease as this is a completely new pathway involving tumor-infiltrating macrophages and NK cells, which bypasses the PD-1/PD-L1 and CTLA4/CD80 pathways. Additionally, combination CD47 blockade with anti-CD19/CD20 suggests a synergistic effect. At the time of this review, there are numerous active studies evaluating all anti-CD47 approaches either as single-agent or in combination for many hematologic malignancies as outline in **Table 12**.

**Table 11:** Published trials evaluating anti-CD47 therapy.

Name/Code	Year	Phase	Disease/ Status	Sample Size	Intervention	Response	Duration	Ref #
NCT02953509	2018	I	R/R DLBCL R/R FL	22	Magrolimab + Rituximab	Combined ORR: 50% CR: 36% DLBCL ORR: 40% CR: 33% FL ORR: 71% CR: 43%	91% mDOR (DLBCL): 6.2 mo 91% mDOR (FL): 8.1 mo	(137)
NCT03248479	2020	I	ir/hrMDS Unfit AML	68	Magrolimab + AZA	MDS ORR: 91% CR: 42% CR (6-mo): 56% AML ORR: 64% CR: 40% TP53 AML CR + CRi: 75% CR: 42% CRi: 33%	mDOR: not reached 6mo DOR (MDS): 91% 6mo DOR (AML): 100% 6mo OR (MDS): 100% 6mo OR (TP53 AML): 91%	(138)
NCT02367196	2019	I	R/R NHL (CD20+)	24	CC-90002 + Rituximab	D/c due to PD or Death: 20 ORR: 13%	mDOR: 3.9 mo	(139)
NCT02641002	2022	I	hrMDS R/R AML	28	CC-90002	All patients d/c due to PD or Death		(140)
NCT03934814	2021	I	R/R NHL (CD20+)	8	Lemzoparlimab + Rituximab	ORR: 57%	median time to response: 2 mo	(142)
NCT02663518	2021	I	CTCL	24	TTI-621	ORR: 20%		(145)
NCT02890368	2021	I	R/R MF, SS	66	Intralesional TTI-621	ORR: 90% 80% saw response in distal lesions		(146)
NCT03530683	2021	I	R/R B-/ T-NHL	42	TTI-622	ORR: 33%		(147)
ASPEN-01	2020	I	R/R NHL (CD20+)	33	ALX148 + Rituximab	ALX148 at 10mg/kg ORR: 40.9% ALX148 at 15mg/kg ORR: 63.6%		(148)
ChiCTR1900024904	2021	I	R/R NHL R/R HL	14	IMM01	1 FL with CR 1 FL with SD 1 HL with PR 1 HL with SD 1 MZL with SD 1 AITL with SD		(149)

R/R Relapsed/Refractory, DLBCL Diffuse Large B-Cell Lymphoma, FL Follicular Lymphoma, ORR Objective Response Rate, CR Complete Response, mDOR Median duration of Response, ir/hrMDS Intermediate/High-Risk Myelodysplastic Syndrome, AML Acute Myeloid Leukemia, AZA Azacitidine, CRi Complete Response with Incomplete Count Recovery, OR Overall Survival, NHL Non-Hodgkin Lymphoma, PD Progressive Disease, SD Stable Disease, PR Partial Response, CTCL Cutaneous T-Cell Lymphoma, MF Mycosis Fungoides, SS Sezary Syndrome, HL Hodgkin Lymphoma, MZL Marginal Zone Lymphoma, AITL Angioimmunoblastic T-Cell Lymphoma.

CC-90002 (anti-CD47 antibody). TTI-621 (SIRPαIgG1 fusion protein). TTI-622 (SIRPαIgG4 fusion protein). ALX148 (SIRPαIgG1 fusion protein). IMM01 (SIRPαIgG1 fusion protein).

**Table 12:** Current clinical trials evaluating anti-CD47 therapy.

NCT Number	Phases	Disease Focus	Interventions
NCT04338659	I	Lymphomas	IBI322
NCT03763149	I	Lymphomas	IBI188
NCT05567887	I	Lymphomas, MM	Maplirpacept
NCT03530683	I	Lymphomas, MM, AML, DLBCL	Maplirpacept
NCT05896774	I	NHL, MM	Maplirpacept
NCT05263271	I	MDS, AML	Gentulizumab
NCT05221385	I	NHL	Gentulizumab
NCT05293912	I	Lymphomas	SG2501
NCT04806035	I	NHL, CLL, SLL	TG-1801 + Ublituximab
NCT03804996	I	Lymphomas	TG-1801 + Ublituximab
NCT03013218	I	NHL	ALX148
NCT05892718	I	R/R NHL	HCB101
NCT05266274	I	R/R AML s/p HCT	CD47 mAb + Azacitidine after alloHCT
NCT02678338	I	MDS, AML	Magrolimab
NCT03527147 (PRISM)	I	NHL, DLBCL	Magrolimab + Rituximab + Acalabrutinib
NCT05823480	I	MDS, AML	Magrolimab + Azacitidine after alloHCT
NCT04599634	I	NHL, CLL	Magrolimab + Obinutuzumab + Venetoclax
NCT05025800	I/II	Aggressive B-NHL	ALX148 + Lenalidomide + Rituximab
NCT04755244	I/II	AML	ALX148 + Azacitidine + Venetoclax
NCT04980885	I/II	AML	AK117 + Azacitidine
NCT05833984	I/II	HL	IMM01 + Tislelizumab
NCT05140811	I/II	MDS, AML	IMM01 + Azacitidine
NCT05805943	I/II	B-NHL	IMM0306
NCT05771883	I/II	B-NHL	IMM0306 + Lenalidomide
NCT05189093	I/II	R/R Lymphomas	HX009
NCT04853329	I/II	B-NHL	CPO107
NCT05626322	I/II	DLBCL	Maplirpacept + Tafasitamab + Lenalidomide
NCT05896163	I/II	DLBCL	Glofitamab + Maplirpacept after Obinutuzumab
NCT04435691	I/II	R/R AML	Magrolimab + Azacitidine + Venetoclax
NCT02953509	I/II	NHL	Magrolimab + Rituximab, Magorlimab + R-GemOx
NCT05367401	I/II	MDS, AML	Magrolimab + Azacitidine + Sabatolimab
NCT04541017	I/II	R/R Mycosis Fungoides, Sezary Syndrome	Magrolimab + Mogamulizumab
NCT05507541	II	R/R HL, R/R NHL	TTI-621 or TTI-622 with Pembrolizumab
NCT04788043	II	R/R HL	Magrolimab + Pembrolizumab
NCT05929716	II	DLBCL	Magrolimab + Rituximab + Radiation as bridge to CAR-T
NCT05829434	II	MDS, AML	Magrolimab with 7 + 3 or CPX-351
NCT04778397	III	TP-53 mutated AML	Magrolimab + Azacitidine vs. Venetoclax + Azacitidine
NCT05079230 (ENHANCE)	III	AML	Magrolimab + Azacitidine + Venetoclax vs Azacitidine + Venetoclax

MM Multiple Myeloma, MDS Myelodysplastic Syndrome, AML Acute Myeloid Leukemia, NHL Non-Hodgkin Lymphoma, DLBCL Diffuse Large B-Cell Lymphoma, HL Hodgkin Lymphoma, R-GemOx Rituximab + Gemcitabine + Oxaliplatin, MF Mycosis Fungoides, SS Sezary Syndrome, R/R Relapsed/Refractory, HCT Hematopoietic Stem Cell Transplant

IBI322 (bispecific anti-CD47/PD-L1 antibody). IBI188 (anti-CD47 antibody). SG2501 (bispecific anti-CD47/CD38 antibody). TG-1801 (bispecific anti-CD47/CD19 antibody). ALX148 (SIRPαIgG1 fusion protein). HCB101 (SIRPαIgG4 fusion protein). AK117 (anti-CD47 antibody). IMM01 (SIRPαIgG1 fusion protein). IMM0306 (bispecific anti-CD47/CD20 antibody). HX009 (bispecific anti-CD47/PD-1 antibody). CPO107 (bispecific anti-CD47/CD20 antibody). TTI-621 (SIRPαIgG1 fusion protein). TTI-622 (SIRPαIgG4 fusion protein).

### NKG2A

Natural-killer group 2 member A (NKG2A) is an inhibitory receptor on both NK cells and T cells that binds to non-classical MHC-I (HLA-E) and induces a “self-recognition signal” to allow malignant cells to evade cytotoxicity. HLA-E is known to be overexpressed in various malignancies including DLBCL, MM, and AML. Monalizumab is an anti-NKG2A antibody which has shown activity in combination with Cetuximab for other solid tumor types, but studies have yet to show efficacy in hematologic malignancies. One study assessed Monalizumab in combination with ibrutinib for treatment of r/r CLL, but the study had been terminated by the sponsor (NCT02557516). More studies are needed to evaluate the utility of anti-NKG2A antibody use in hematologic malignancies.

### KIR

Killer Cell immunoglobulin-like receptor (KIR) is another NK cell inhibitory receptor that binds classical MHC-1 and leads to immune evasion. IPH2102 (1-7F9) is an anti-KIR mAb that has been studied in both r/r MM and AML after first CR. These Phase I studies have at least shown a tolerable safety profile, but minimal or no ORR (152, 153). The previously mentioned study by Armand et al. evaluated the recombinant anti-KIR mAb, Lirilumab, in combination with Nivolumab in multiple hematologic malignancies, but it was concluded that there was no benefit to the addition of anti-KIR to Nivolumab (12). Available data thus far is limited on the efficacy of anti-KIR ICI therapy, but current trials are underway. NCT01256073 is assessing safety and tolerability of IPH2101 in older AML patients over 60 years of age who are not transplant eligible. The EFFIKIR Trial (NCT01687387) is evaluating efficacy of Lirilumab as maintenance therapy for elderly AML patients after first CR. Lastly, NCT02481297 is assessing the safety of Lirilumab in combination with Rituximab in patients with r/r CLL. Additionally, genetic variability in KIRs due to allelic polymorphisms has been implicated in the variability of patients’ responses in allo- and haptoidentical HCT (154). Thus, a number of current studies aim to better understand the importance of donor selection based on HLA and KIR profile matching, which could open up more avenues toward the therapeutic addition of anti-KIR ICI.

## Concluding remarks

ICI therapy has shown to be beneficial in a multitude of solid malignancies and more recently in hematologic malignancies. Hodgkin Lymphoma has seen the most benefit from ICI-based therapy, mainly in the relapsed and refractory setting after autologous stem cell transplant, but recent and current studies have shown promising data to suggest a role of ICI in earlier lines of therapy as well. Most notably, Nivolumab showed superior PFS when compared to Brentuximab vedotin when combined with first-line AVD for advanced stage cHL. Data for Non-Hodgkin Lymphomas have been less impressive, but ICI may have a role specifically in the treatment of Richter-Transformed DLBC and PCNSL. Though data to support ICI as either monotherapy or in combination is limited, more studies are looking into ICI as a sensitizing or synergistic agent with other therapies for NHL. ICI therapy has been implicated in the treatment of PCNSL, especially given the association with EBV reactivation and induction of PD-L1 expression in these tumors. T cell Lymphoma and Leukemia has seen mixed responses to ICI, though Sintilimab has had promising benefits specifically in ENKTL. ICI-containing combination regimens may also show improved activity in treating PTCL. Unfortunately, data thus far have shown limited benefit for ICI in Multiple Myeloma, suggesting that polymorphisms within the PD-1 and CTLA-4 genes may play a role in the variability of responses among all hematologic malignancies. MDS and AML have had modest benefit with ICI, most notably in combination with HMA. More promising is the activity seen with anti-CD47 antibody therapy (Magrolimab) in the treatment of high-risk MDS and TP-53 mutated AML. In contrast, ALL has not seen as robust benefit with ICI, though there may be some activity when used in combination with SOC therapies.

Aside from ICI as a component of primary systemic therapy, there seems to be a role in augmenting the efficacy of cellular therapies. When given after CAR-T therapy, ICI has been shown to induce re-expansion of the CAR-T population and may be useful as adjunctive therapy to maintain CAR-T anti-tumor activity. In the setting of allogeneic HCT, the risk of worsened GVHD from ICI is at least mitigated with the use of posttransplant cyclophosphamide. Thus, ICI is a viable treatment option both before and after cellular therapies.

PD-1/PD-L1 and CTLA-4 remain the primary targets for ICI, but recent research has revealed other checkpoint molecules of potential clinical significance. LAG-3, TIM-3, and TIGIT blockade have shown promising activity in many hematologic malignancies. Most exciting is the macrophage checkpoint inhibitor, CD47, in which blockade in combination with HMA has shown significant activity against high-risk MDS and TP-53 mutated AML. Lastly, NK/T cell checkpoint molecules such as NKG2A and KIR may also play a role in tumor immune evasion and serve as potential alternative therapeutic targets.

Overall, ICI continues to show great potential in the treatment of hematologic malignancies as either monotherapy, in combination with other systemic therapies, or as a synergistic agent. Further discovery of new novel checkpoint molecules yields more alternative approaches to enhance the immune system’s recognition of tumor cells and promises of improved outcomes.

## Author contributions

AT: Conceptualization, Data curation, Investigation, Methodology, Project administration, Visualization, Writing – original draft, Writing – review & editing. DL: Conceptualization, Data curation, Investigation, Visualization, Writing – original draft. JT: Conceptualization, Project administration, Supervision, Writing – review & editing.
